# The Biological Activity of AAV Vectors for Choroideremia Gene Therapy Can Be Measured by *In Vitro* Prenylation of RAB6A

**DOI:** 10.1016/j.omtm.2018.03.009

**Published:** 2018-03-28

**Authors:** Maria I. Patrício, Alun R. Barnard, Christopher I. Cox, Clare Blue, Robert E. MacLaren

**Affiliations:** 1 Nuffield Laboratory of Ophthalmology, Nuffield Department of Clinical Neurosciences, University of Oxford, Oxford, UK; 2 National Institute for Health Research (NIHR) Oxford Biomedical Research Centre (BRC), Oxford, UK; 3 Oxford Eye Hospital, Oxford University Hospitals NHS Foundation Trust, Oxford, UK; 4 Nightstar Therapeutics, Wellcome Gibbs Building, 215 Euston Road, London, UK

**Keywords:** AAV gene therapy, choroideremia, prenylation, potency assay

## Abstract

Choroideremia (CHM) is a rare, X-linked recessive retinal dystrophy caused by mutations in the *CHM* gene. *CHM* is ubiquitously expressed in human cells and encodes Rab escort protein 1 (REP1). REP1 plays a key role in intracellular trafficking through the prenylation of Rab GTPases, a reaction that can be reproduced *in vitro*. With recent advances in adeno-associated virus (AAV) gene therapy for CHM showing gene replacement to be a promising approach, an assay to assess the biological activity of the vectors is of the uttermost importance. Here we sought to compare the response of two Rab proteins, RAB27A and RAB6A, to the incorporation of a biotinylated lipid donor in a prenylation reaction *in vitro*. First, we found the expression of REP1 to be proportional to the amount of recombinant AAV (rAAV)2/2-REP1 used to transduce the cells. Second, prenylation of RAB6A appeared to be more sensitive to REP1 protein expression than prenylation of RAB27A. Moreover, the method was reproducible in other cell lines. These results support the further development of a prenylation reaction using a biotinylated lipid donor and RAB6A to assess the biological activity of AAV vectors for CHM gene therapy.

## Introduction

Choroideremia (CHM) is an X-linked retinal dystrophy that leads to degeneration of the retinal pigment epithelium (RPE) and the photoreceptors of the eye. Afflicted males typically exhibit night blindness during teenage years and progressive loss of peripheral vision during the 20s and 30s, which can result in complete blindness by the 40s.^1^ Female carriers usually maintain good vision throughout life;^2^ however, more severe female phenotypes have been reported.^3^

CHM is caused by mutations in the *CHM* gene, which encodes for Rab escort protein 1 (REP1). REP1 is ubiquitously expressed in all human cells and is involved in the C terminus posttranscriptional modification of Rab GTPases, the largest family within the Ras-like GTPase superfamily. This modification, known as prenylation, is catalyzed by the Rab geranylgeranyl transferase (RGGT or GGT-II) and involves the covalent attachment of one or more C20 (geranylgeranyl) isoprenoid groups to a cysteine residue within a “prenylation motif.” REP1 assists by either presenting the unprenylated Rabs to the GGT-II and/or escorting the prenylated Rabs to their destination membrane, where they play a role in vesicle trafficking.^4^, ^5^, ^6^

The CHM-like gene (*CHML*) encodes for REP2. REP2 shares 95% of its amino acid sequence with REP1, and studies have shown that REP2 can compensate for REP1 deficiency in most cells of the body.^7^, ^8^ However, REP2 is unable to fully compensate for REP1 deficiency in the eye for reasons that are not yet clear. In patients with CHM, the prenylation of Rab GTPases in the eye is affected, which causes cellular dysfunction and ultimately cell death.^8^

CHM may be treated by providing functional copies of the *CHM* gene to the affected cells of the eye. Specifically, a recombinant adeno-associated virus (rAAV) vector encoding *CHM* was delivered subretinally to six CHM patients.^9^ The 6-month results of this clinical trial showed improvements in visual acuity in two patients, which was sustained 3.5 years after treatment.^10^ Eight more patients were treated in this trial, and, over the long term, there was an improvement in vision in treated eyes compared with untreated eyes in the first 14 patients undergoing retinal gene therapy (MacLaren et al., 2018, ARVO Annual Meeting, abstract). As AAV gene therapy of CHM is becoming a clinical reality with several ongoing studies (NCT01461213, NCT02407678, NCT02671539, NCT02553135, NCT02077361, NCT02341807), there is a need for reliable and sensitive *in vitro* assays to determine the biological activity of rAAV2/2-REP1.

A prenylation reaction can be reproduced *in vitro* to test for REP1 biological activity.^11^ Previously described methods for assaying REP1 following viral transduction have used RAB27A as a substrate in a prenylation reaction.^12^, ^13^, ^14^, ^15^ This has likely followed from the fact that this protein was first identified in the cytosolic fraction of CHM lymphoblasts in 1995.^16^

This study is the first to compare RAB27A with another Rab protein, RAB6A, as substrates in a prenylation assay *in vitro* with the aim of developing more robust and sensitive assays for assessing the biological activity of AAV vectors for CHM.

## Results

**Figure 1:**
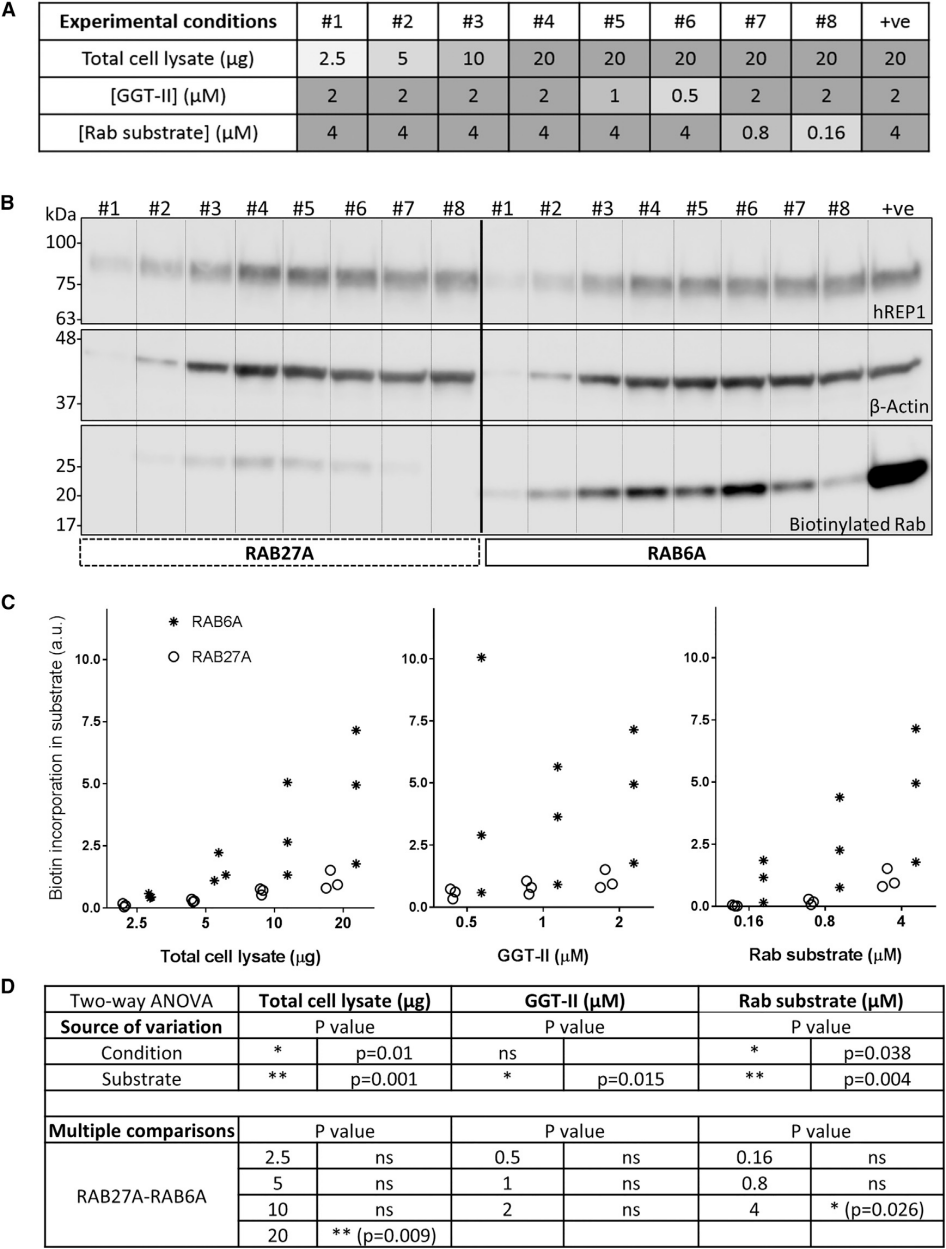
Both RAB27A and RAB6A Are Subject to Prenylation by Endogenous REP1 from a HEK293-Cell Lysate (A) Summary table of experimental conditions (1–8) used in prenylation reactions *in vitro* regarding the amount of total cell lysate (2.5, 5, 10, and 20 μg), concentration of GGT-II (0.5, 1, and 2 μM), and concentration of Rab substrate (RAB27A or RAB6A) (0.16, 0.8, and 4 μM). Positive control (+ve): cell lysate spiked with recombinant fish REP1 (25 nM). (B) Protein expression (human REP1 and β-actin) and biotin incorporation detected in prenylation reaction products following SDS-PAGE and western blot analysis (one example out of three independent experiments). (C) Plots for condition sets assessing biotin incorporation in both RAB27A and RAB6A when different amounts of total cell lysate, concentration of GGT-II, or concentration of Rab substrate were used (n = 3). (D) Summary table of statistical analysis performed in the datasets in (C). Two-way ANOVA tests were run independently for each condition (total cell lysate, concentration of GGT-II, or concentration of Rab substrate) with “condition” and “substrate” as factors. The p values and the significance of each test, as well as the Bonferroni’s multiple comparison test for comparison of RAB27A with RAB6A, are given in detail.

A method for the detection of small GTPases *in vitro* was established approximately 20 years ago using radiolabeled-prenyl donors.^11^ More recently, radiolabeling was replaced by either a fluorophore or a biotin group.^17^ Both approaches involved the use of a cultured cell lysate because REP1 is ubiquitously expressed in all cells and tissues. In this study, protein incorporation of biotin-containing isoprenoids (biotin-labeled geranyl pyrophosphate [B-GPP]) was used to detect prenylated proteins due to their superior sensitivity to fluorescence-based methods.^17^ The first step in establishing an assay of this nature was to optimize the prenylation reaction conditions to detect endogenous REP1 activity (Figure 1). The experimental conditions tested are depicted in Figure 1A and include the amount of total cell protein (2.5, 5, 10, and 20 μg), concentration of GGT-II (0.5, 1, and 2 μM), and concentration of Rab substrate, either RAB27A or RAB6A (0.16, 0.8, and 4 μM). Three separate cell lysates were used to run the three independent experiments. The reaction products were subjected to western blot analysis, of which one example is shown in Figure 1B. The positive control (+ve) reaction was run with 2 μM of GGT-II and 4 μM of RAB6A and spiked with recombinant fish REP1 (25 nM). The band intensity for biotin incorporation in the positive control well (Figure 1B, right-hand side) proves that all substrates involved were in appropriate conditions and the reaction was run successfully. In all three experiments, we observed that both substrates were prenylated *in vitro* by endogenous REP1 in a dose-dependent manner as measured by the biotin incorporation (Figures 1B and 1C), suggesting that both could be used to assess the biological activity of rAAV2/2-delivered REP1. As for statistical data analysis, three independent two-way ANOVA were run to compare the biotin incorporation between RAB27A and RAB6A for each of the conditions tested. The two-way ANOVA with “condition” (total cell lysate) and “substrate” as factors revealed both factors were significantly contributing to the source of variation in conditions 1–4 (Figure 1D; n = 3; p = 0.01 and p = 0.001, respectively). However, a Bonferroni’s multiple comparison test for biotin incorporation found that RAB6A incorporated significantly more B-GPP than RAB27A when 20 μg of total cell lysate was used (Figure 1D; p = 0.009). The same approach was used for analyzing the impact of both the concentration of GGT-II (conditions 4–6) and the concentration of Rab substrate (conditions 4, 7, and 8). The two-way ANOVA analysis with GGT-II concentration as “condition” revealed that only the “substrate” contributes to the source of variation in this case (Figure 1D; n = 3; p = 0.015). The Bonferroni’s multiple comparison tests for biotin incorporation found no statistically significant differences between RAB27A and RAB6A when the concentration of GGT-II varied (Figure 1D; not significant [ns]). Regarding the Rab substrate concentrations (conditions 4, 7, and 8), a two-way ANOVA analysis showed both “condition” and “substrate” to be contributing factors to the source of variation (Figure 1D; n = 3; p = 0.038 and p = 0.004, respectively). A Bonferroni’s multiple comparison test for biotin incorporation found RAB6A to have incorporated significantly more B-GPP than RAB27A when 4 μM of Rab substrate was used (p = 0.026). These data shows that the use of different Rab substrates significantly influenced the results obtained in all conditions tested. Moreover, the concentration of GGT-II in reaction is the least contributing factor for the biotin incorporation in the substrate, possibly because it was used in excess. Based on the evidence collected in this optimization step, we decided to use 20 μg of total cell lysate, 2 μM of GGT-II, and 4 μM of Rab substrate as standard conditions in further investigations about differences in biotin incorporation in RAB27A and RAB6A using HEK293 cells.

**Figure 2:**
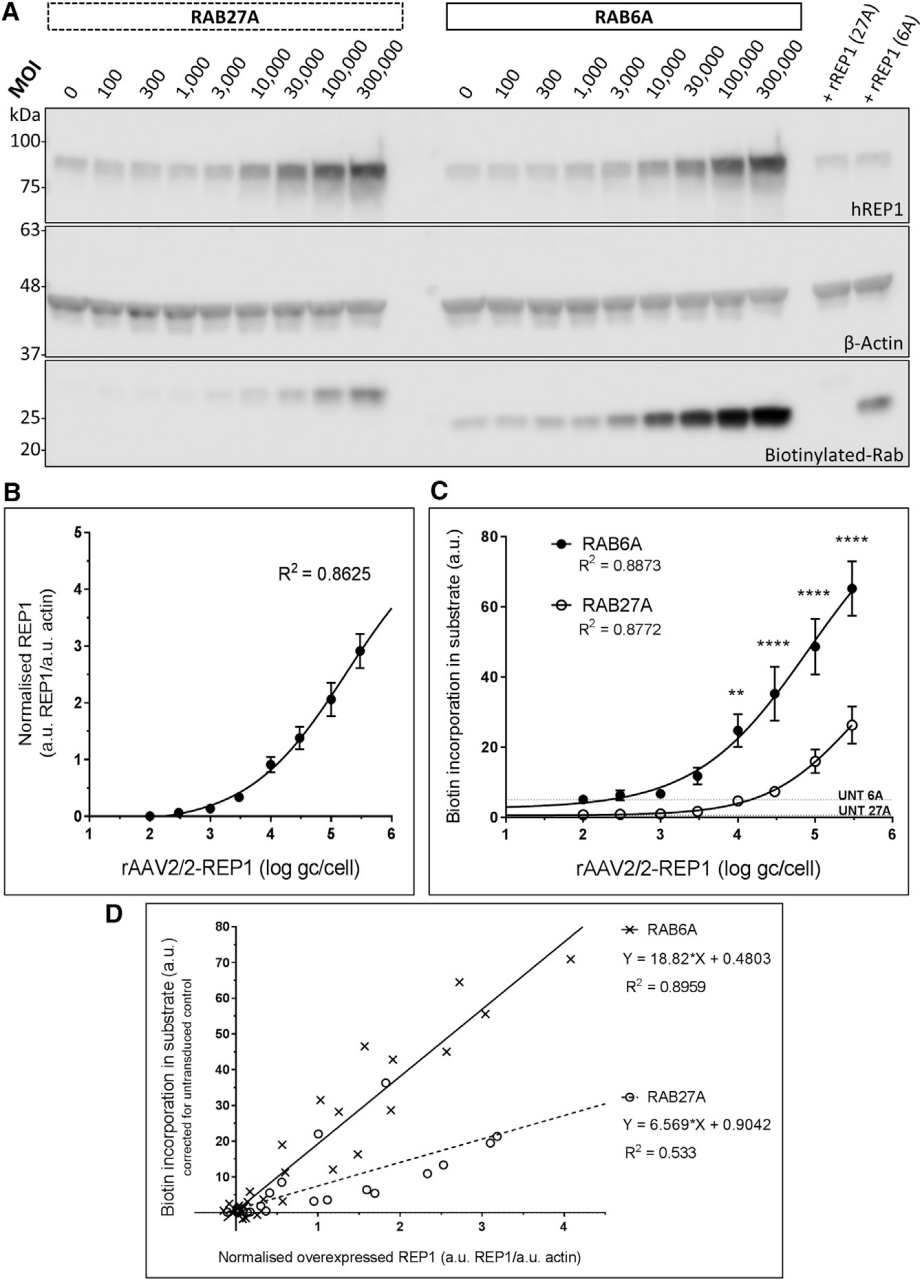
RAB6A Is More Sensitive Than RAB27A to Assess the Biological Activity of Human REP1 following rAAV2/2 Transduction of HEK293 Cells (A) The HEK293 cells were transduced with increasing MOI of rAAV2/2-REP1 (100, 300, 1,000, 3,000, 10,000, 30,000, 100,000, and 300,000). Protein expression (human REP1 and β-actin) and biotin incorporation were detected in prenylation reaction products (20 μg) following SDS-PAGE and western blot analysis (representative image of three independent experiments). (B) Nonlinear regression plot of normalized REP1 (corrected for the corresponding actin levels) *per* rAAV2/2-REP1 (log gc/cell). Data were analyzed using a sigmoidal four-parameter fit (95% confidence interval; R^2^ = 0.8625). Symbols are mean of 6 replicates ± SEM. C) Nonlinear regression plots of biotin incorporation *per* MOI of rAAV2-REP1 (log gc/cell). Data was analyzed using a sigmoidal four-parameter fit (95% confidence interval; R^2^ = 0.8873 for RAB6A; R^2^ = 0.8772 for RAB27A). Symbols represent the mean of three replicates ± SEM. RAB6A showed statistically significant higher incorporation of biotin than RAB27A at MOI 10,000 (**p = 0.01) and 30,000, 100,000, and 300,000 (****p ≤ 0.0002) (two-way ANOVA with Bonferroni’s multiple comparison test). (D) Linear regression plots of biotin incorporation in substrate corrected for the untransduced control against the normalized overexpressed REP1 for RAB6A (R^2^ = 0.8959, Y = 18.82 × X + 0.4803) and RAB27A (R^2^ = 0.533, Y = 6.569 × X + 0.9042).

Next, we tested both Rab proteins as substrates in a scenario of *CHM* gene augmentation. Three independent experiments were run where HEK293 cells were transduced with rAAV2/2-REP1 at a range of increasing MOIs (defined as the number of genome copies/cell [gc/cell]) (100, 300, 1,000, 3,000, 10,000, 30,000, 100,000, and 300,000) (Figure 2). The prenylation reaction products were analyzed simultaneously in each experiment, using actin as a loading control; a representative western blot is shown in Figure 2A. Two positive control reactions (one for each Rab substrate) were run in parallel with recombinant fish REP1 (25 nM) spiked in the untransduced cell lysate.

The first observation is that the amount of REP1 detected by western blot correlates to the amount of viral particles added to the cells (Figure 2A, top panel): the band density for REP1 increases as the MOI increases. We plotted the normalized REP1 band density (to corresponding actin band density) against the MOI (log scale) using a 4-parameter logistic (4-PL) regression model (Figure 2B). This model takes into consideration that cells are a biologically limited system in this experiment, where increasing MOI will saturate the system at some point and cease REP1 production. The regression model was run with no constrains (R^2^ = 0.8625) and predicted the best-fit value for the top of the curve to be 5.191 a.u. of normalized REP1. The log(IC_50_) for this fit was 5.255 a.u., corresponding to a MOI of approximately 170,000 gc/cell, which is within the range that we tested.

The second observation refers to the biotin incorporation in the Rab substrate (Figure 2A, bottom panel). Following the same rationale as for REP1 in Figure 2B, the biotin incorporation in both substrates (as measured by the band density) was plotted against the MOI of rAAV2/2-REP1 used for transduction (Figure 2C). The baseline value obtained for each Rab in the untransduced samples (average of three independent runs) is represented by the horizontal dotted line (RAB6A, 5.009 ± 1.25 a.u.; RAB27A, 0.577 ± 0.19 a.u.). A 4-PL regression model was run for each Rab substrate, without any constrains, and both R^2^ are shown in Figure 2C (RAB27A, R^2^ = 0.8772; RAB6A, R^2^ = 0.8873). The best-fit prediction for the RAB6A top of the curve is 92.83 a.u., with a log(IC_50_) of 4.912, corresponding to a MOI of approximately 80,000 gc/cell. For RAB27A, the top of the curve is predicted to be 53.8 a.u., with a log(IC_50_) of 5.514, corresponding to a MOI of approximately 320,000 gc/cell. The differences between the log(IC_50_) values for each Rab substrate are indicative of their sensitivity in this assay, and incorporation of biotin in RAB6A can be detected over a wider range than in RAB27A, which displays a lower slope and limit of detection. These findings were reinforced by the two-way ANOVA run in the same dataset, with “MOI” and “substrate” as factors, and both were found to be significant (n = 3; p < 0.0001). Moreover, Bonferroni’s multiple comparison tests for biotin incorporation in the substrate at each tested MOI revealed that such incorporation was significantly higher in RAB6A than RAB27A at the MOI of 10,000 (p = 0.01) and 30,000, 100,000, and 300,000 (p ≤ 0.0002). These results reinforce the superiority of RAB6A in incorporating biotin at a given MOI of rAAV2/2-REP1.

We then plotted each value of the biotin incorporation in substrate, corrected for the corresponding untransduced sample, against the normalized overexpressed REP1 (Figure 2D). The resultant linear regression analysis shows that incorporation of biotin on RAB6A *per* unit of REP1 is higher than for RAB27A (Y = 18.82 × X + 0.4803 versus Y = 6.569 × X + 0.9042, respectively). The RAB6A dataset also shows a better fit to the regression (R^2^ = 0.8959 versus R^2^ = 0.533 for RAB27A). Altogether, our data show that RAB6A is more sensitive to use as a substrate to measure the biological activity of rAAV2/2-REP1.

**Figure 3:**
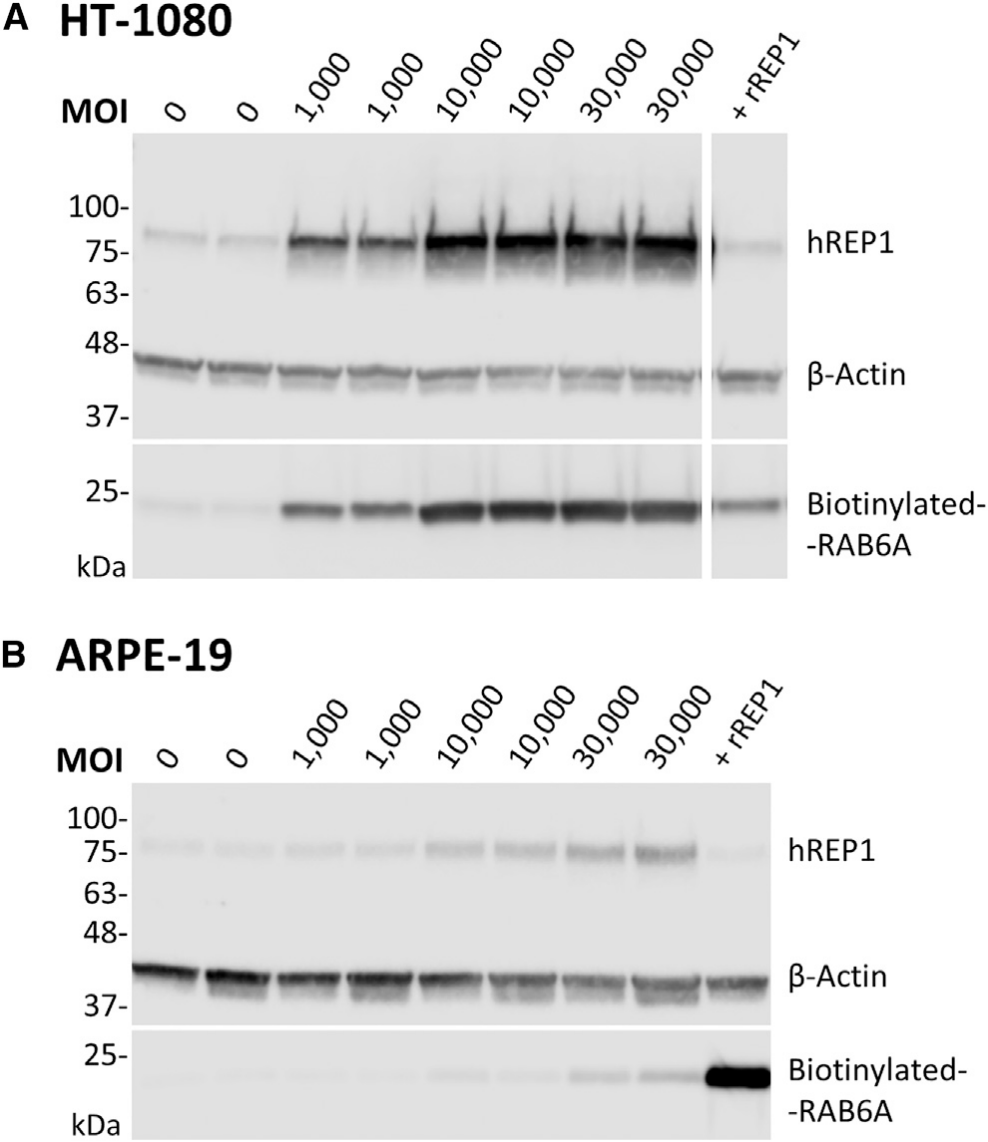
RAB6A Validation as a Substrate for *In Vitro* Prenylation by Other Cell Lines Protein expression (human REP1 and β-actin) and biotin incorporation were detected in prenylation reaction products following cell transduction, SDS-PAGE, and western blot analysis (two replicates in one experiment). HT-1080 cells (A) and ARPE-19 cells (B) were transduced with rAAV2/2-REP1 (MOI 1,000; 10,000; and 30,000 gc/cell), and prenylation reactions were prepared with 20 μg and 10 μg of total protein, respectively. Positive controls (+ rREP1) were prepared using untransduced cell lysate spiked with a recombinant fish REP1 protein (25 nM for HT-1080; 11 nM for ARPE-19).

To further confirm that RAB6A use as a substrate in an *in vitro* prenylation assay was applicable to other cell lines, HT-1080 (human fibrosarcoma) and ARPE-19 (human RPE) were transduced with rAAV2/2-REP1 in a similar manner for a qualitative analysis. In both cases, a representative MOI of 1,000, 10,000, and 30,000 gc/cell was used to transduce two wells (replicates) in one single experiment. A positive control was run in parallel with recombinant fish REP1 spiked in each untransduced cell lysate (25 nM for HT-1080; 11 nM for ARPE-19). The prenylation products were subjected to western blot analysis, and the results are shown in Figure 3. We observed a correlation between the MOI used for transduction, the expression of REP1, and the incorporation of biotin in RAB6A, as we did for HEK293 cells (Figures 3A and 3B). However, REP1 levels detected for ARPE-19 and corresponding biotinylated-RAB6A were overall lower than for HT-1080 and HEK293 cells. This fact is justified by the larger size of ARPE-19 cells, which required a reduced number of cells seeded in each well and volume restrictions to the total amount of cell lysate that could be loaded in the gel. Nevertheless, this experiment shows that our method can be reproduced in other cell lines.

## Discussion

For the first time, we report the use of a biotinylated lipid donor and a Rab substrate to measure the biological activity of rAAV2/2-delivered REP1 *in vitro*. The aim is to provide a reproducible and sensitive *in vitro* test for assessing the biological activity of AAV gene therapy vectors for CHM.

The first use of *in vitro* prenylation of RAB27A to measure the biological activity of REP1 delivered by a vector was reported in 2012.^12^ In their work, Tolmachova et al.^12^ used a radioactive-labeled prenyl donor to confirm the functionality of the *CHM* transgene delivered to cells by an HIV-based lentiviral vector. Later on, the same group and another reported the use of RAB27A to test AAV vectors for REP1 (rAAV2/2-REP1^13^ and rAAV2/8.CBA.hCHM^15^).

RAB27A was first identified among the subset of Rab proteins found under-prenylated in CHM lymphoblasts.^16^ The same study concluded that RAB27A has a lower affinity for REP2 than for REP1 compared with other Rab proteins, implying that it could not compete efficiently for prenylation in the absence of REP1.^16^ However, it was later reported that RAB27A binds equally well to REP1 and REP2.^18^ Instead, RAB27A is likely to accumulate unprenylated proteins due to the fact that the RAB27A-REP1 complex was actually found to have a higher affinity for GGT-II than RAB27A-REP2.^18^ Furthermore, RAB27A was shown to have one of the slowest rates of GTP hydrolysis^18^ and one of the slowest prenylation rates among Rab proteins.^19^ Altogether, these results point to under-prenylation of RAB27A as one of the molecular causes of degeneration of RPE cells in CHM,^16^, ^18^, ^19^ although these and other authors suggest other cellular perturbations may contribute to the CHM phenotype.^4^

As defined by the US Food and Drug Administration (FDA), a biological assay is a “quantitative assay that measures the activity of the product related to its specific ability to effect a given result.”^20^ The recent development of biotinylated lipid donors has paved the way for simpler and more sensitive methods of assessing prenylation *in vitro*.^17^ The use of biotin-labeled prenyl donors has now been reported for detection of unprenylated Rab protein levels in HeLa,^19^ lymphoblasts,^19^ fibroblasts,^21^ and induced pluripotent stem cell (iPSC)-derived RPE^22^ cells. Moreover, biotinylated lipid donors have also been used to quantify the levels of prenylated Rab substrates in HeLa cells,^19^ corroborating its potential benefit in a biological assay.

HEK293 cells were the cell line of choice for this study because they were commercially available from a certified cell line provider and a master cell bank compliant with current good manufacturing processes (cGMP). These characteristics will favor the development a potency test for gene therapy products according to the FDA recommendations. Moreover, due to REP1 ubiquitous expression, and in the absence of a REP1-deficient stable cell line, there will always be an endogenous level of REP1 present. Therefore, an assay that maximizes the measured response is the most beneficial to distinguish between the endogenous and vector transgene-expressed REP1. Evidence from the literature prompted us to use RAB27A, for the reasons aforementioned, and RAB6A. According to Köhnke et al.,^19^ RAB6A is the exact opposite of RAB27A regarding the prenylation rate. It is at the top hierarchy of Rab protein prenylation rate and will therefore provide a more sensitive readout of increased activity. For this reason, we sought to compare it with RAB27A for use as a substrate in a biological activity assay. Our first experiment showed that both substrates could be used to measure prenylation activity in untransduced cells. Moreover, the fact that the band density obtained with RAB6A was constantly higher than RAB27A for the same conditions tested corroborates the published literature.^19^ We then tested how both substrates would behave in response to rAAV2/2-delivered REP1. We believe that the statistical best-fit for the relationship between REP1 expression and MOI is not linear but rather logarithmic, as there is a limit to the extent to which cells can be transduced by rAAV. The sigmoidal-shaped curve implies there is a limit for the amount of REP1 expressed from an exogenously-delivered transgene that can be measured using this protocol, which we have not reached in this experiment. This is also true for biotin incorporation in the Rab substrate. The data analysis supports RAB6A as a more efficient substrate to measure biotin incorporation because its range is wider and steeper than RAB27A. The linear regression analysis run on both datasets shows that RAB6A has higher biotin incorporation within the range where normalized REP1 is linear. Therefore, RAB6A is the substrate that predicts more accurately how much biotin is incorporated *per* unit of overexpressed REP1, a key feature when testing its biological activity.

The use of RAB6A was further assessed in other cell lines. HT-1080 cells have been used before to test a lentiviral construct delivering REP1^12^ and to confirm REP1 expression following the use of rAAV2/2-REP1 in a CHM gene therapy trial (NCT01461213).^9^ ARPE-19 cells were selected for their similarity to the target cell type of CHM gene therapy. Both cell lines responded as HEK293 cells regarding the incorporation of biotin in RAB6A following an *in vitro* prenylation protocol, confirming this assay is reproducible and does not appear to be cell-type specific.

### Conclusions

Altogether, our data show that *in vitro* prenylation of RAB6A is a robust method to test REP1 activity following cell transduction with rAAV2/2. Furthermore, we show that RAB6A appears to be more sensitive as a substrate in a potency assay for rAAV2/2-REP1 because it allows detection of minor differences between viral vector batches more accurately. In conclusion, our study brings valuable improvements to the development of an *in vitro* prenylation assay to assess the biological activity of AAV vectors in CHM gene therapy clinical trials.

## Materials and Methods

### rAAV Vector Production

A recombinant AAV2/2 viral vector containing the *CHM* transgene under the control of a CAG promoter^5^ was produced at the Nationwide Children’s Hospital (OH, USA) following a standard protocol^23^ with some modifications. Briefly, HEK293 cells were co-transfected with calcium phosphate, and viral particles were purified from the cell lysates using iodixanol discontinuous centrifugation and heparin chromatography. The viral stock was prepared in formulation buffer (20 mM Tris pH 8.0, 1 mM MgCl_2_, 200 mM NaCl, 0.001% PF68 in water for injections) at a concentration of 4.95E+12 DNA-resistant particles (DRPs)/mL.

### Cell Culture

HEK293 cells (293, #85120602, Culture Collections, Public Health England, Salisbury, UK) were cultured in MEM culture medium. HT-1080 cells (human fibrosarcoma, #85111505, Culture Collections, Public Health England, Salisbury, UK) were cultured in DMEM. ARPE-19 cells (human RPE, #CRL-2302, ATCC via LGC Standards, Middlesex, UK) were cultured in DMEM:F12. MEM culture medium was supplemented with L-glutamine (2 mM). All three culture media were supplemented with penicillin (100 U/mL), streptomycin (100 μg/mL), non-essential amino acids (1%), and 10% fetal bovine serum. Cells were maintained at 37°C in a 5% CO_2_ environment.

### Cell Transduction and Preparation of Total Cell Lysates

For transduction experiments, all cells were seeded in 6-well plates on the day prior to transduction: HEK293, 9.5E+05 cells/well; HT-1080, 4E+05 cells/well; ARPE-19, 2E+05 cells/well. Transduction with rAAV2/2 was performed at a range of MOI (i.e., genome copies/cell), and media were changed 3 days post-transduction (dpt) and every 2–3 days thereafter. Cell lysates were prepared at 5 dpt as follows: cells were washed with PBS and incubated with prenylation buffer (50 mM HEPES, 50 mM NaCl, 2 mM MgCl2, 1 mM DTT, pH 7.5) supplemented with protease inhibitors (cOmplete Mini, Roche, Welwyn, UK) on ice. Cells were scraped into a 1.5 mL tube using a cell scraper, incubated on ice for 15 min, and then disrupted by passing them 20 times through a 26G needle attached to a 1 mL syringe. Cells were centrifuged for 5 min at 1,500 relative centrifugal force (RCF) at 4°C, and the supernatant was transferred to cellulose propionate tubes and centrifuged at 100,000 RCF for 1 hr at 4°C. The supernatant was kept as total cell lysate for prenylation reactions *in vitro*. Total protein content was determined using the Bradford method according to the manufacturer’s instructions (Quick Start Bradford 1x Dye Reagent, Bio-Rad, Hertfordshire, UK), and sample values were extrapolated from a standard curve using a sigmoidal 4-PL regression.

### *In Vitro* Prenylation Assay

The prenylation reactions were set up using total cell lysate (up to 20 μg), recombinant rat GGT-II (2 μM, Jena Biosciences, Jena, Germany), recombinant human Rab protein (RAB27A, Abnova, UK; RAB6A, Jena Biosciences, Jena, Germany), and biotin-labeled geranyl pyrophosphate (B-GPP, 5 μM, Jena Biosciences, Jena, Germany) as lipid donors in prenylation buffer. All reactions were supplemented with fresh guanosine 5′-diphosphate (GDP, 20 μM, Merck Millipore, Watford, UK) and DTT (1 mM, Thermo Fisher Scientific, Loughborough, UK). Positive controls were prepared using untransduced cell lysate spiked with a recombinant REP1 protein (fish His-REP1, Jena Biosciences, Jena, Germany). The reactions were incubated for 2 hr at 37°C and then stopped by addition of Laemmli sample buffer.

### Western Blot Analysis

Reaction products were subjected to SDS-PAGE on 10% pre-cast polyacrylamide gel (Criterion, Bio-Rad, Hertfordshire, UK), transferred to a PVDF membrane (TransBlot Turbo, Bio-Rad, Hertfordshire, UK), and blocked with blocking buffer (PBS+0.1% Tween20 [PBST]+3% BSA) for 45 min. For protein expression, membranes were incubated separately for anti-β-actin (AM4302, Thermo Fisher Scientific, Loughborough, UK; 1:50,000) and anti-human REP1 (MABN52, Merck Millipore, Watford, UK; 1:2,500) primary antibodies in blocking buffer for 1 hr under agitation. Membranes were washed 3× for 7 min with PBST, incubated with horseradish peroxidase (HRP)-labeled secondary antibody for 30 min in blocking buffer (1:10,000), washed again as before, and detected using Clarity ECL (Bio-Rad, Hertfordshire, UK) and an Odyssey Imaging System (LI-COR Biosciences, Cambridge, UK). The incorporation of biotinylated lipid donor into the appropriate Rab substrate was detected by direct incubation with streptavidin-HRP (Thermo Fisher Scientific, Loughborough, UK) for 30 min. Densitometry data analysis was performed using ImageStudio Lite software (LI-COR Biosciences, Cambridge, UK).

### Statistical Analysis

Biotin incorporation in both RAB27A and RAB6A using different experimental conditions was compared using a two-way ANOVA with “substrate” and “condition” as factors (mean of 3 replicates ± SEM). The Bonferroni test was applied to correct for multiple comparisons, with a 95% confidence interval (CI). The normalized REP1 (corrected for corresponding actin levels) was plotted against a log-base-10 transformed MOI of rAAV2/2-REP1 (log gc/cell) and fitted to a 4-PL regression model with 95% CI, no constrains (mean of 6 replicates ± SEM). Biotin incorporation in both substrates was plotted against the MOI of rAAV2/2-REP1 (log gc/cell) and fitted to a 4-PL regression model with 95% CI, no constrains (mean of 3 replicates ± SEM). Biotin incorporation per substrate for each MOI was compared using a two-way ANOVA with “substrate” and “MOI” as factors. The Bonferroni test was applied to correct for multiple comparisons (95% CI). Biotin incorporation in each substrate was plotted against the levels of normalized REP1 (corrected for untransduced control sample) and analyzed by linear regression (95% CI). All statistical analysis was done using Prism 7 for Windows (San Diego, CA, USA).

## Author Contributions

Conceptualization: M.I.P., A.R.B., C.I.C., C.B., R.E.M.; Methodology: M.I.P.; Formal Analysis: M.I.P., A.R.B., C.I.C., C.B., R.E.M.; Investigation: M.I.P.; Writing – Original draft: M.I.P.; Writing – Review & Editing: M.I.P., A.R.B., C.I.C, C.B., R.E.M.; Supervision: A.R.B., R.E.M.; Funding acquisition: R.E.M.

## Conflicts of Interest

M.I.P. and R.E.M. are coinventors on a pending patent for “prenylation assay” filed on behalf of Nightstar Therapeutics, a gene therapy company established by the University of Oxford and based at the Wellcome Trust Building, 215 Euston Road, London NW1 2BE, UK. A.R.B. is a consultant for Nightstar Therapeutics. C.I.C. and C.B. are employees at Nightstar Therapeutics. R.E.M. is a founder and director of Nightstar Therapeutics. R.E.M. receives research funding from Nightstar Therapeutics through the University of Oxford.
